# Edaravone Guards Dopamine Neurons in a Rotenone Model for Parkinson's Disease

**DOI:** 10.1371/journal.pone.0020677

**Published:** 2011-06-03

**Authors:** Nian Xiong, Jing Xiong, Ghanshyam Khare, Chunnuan Chen, Jinsha Huang, Ying Zhao, Zhentao Zhang, Xian Qiao, Yuan Feng, Harrish Reesaul, Yongxue Zhang, Shenggang Sun, Zhicheng Lin, Tao Wang

**Affiliations:** 1 Department of Neurology, Union Hospital, Tongji Medical College, Huazhong University of Science and Technology, Hubei, China; 2 Department of Psychiatry and Harvard NeuroDiscovery Center, Harvard Medical School, Division of Alcohol and Drug Abuse, and Mailman Research Center, McLean Hospital, Belmont, Massachusetts, United States of America; 3 Department of Nuclear Medicine, Union Hospital, Tongji Medical College, Huazhong University of Science and Technology, Hubei Province Key Laboratory of Molecular Imaging, Wuhan, Hubei, China; Institute of Automation, Chinese Academy of Sciences, China

## Abstract

3-methyl-1-phenyl-2-pyrazolin-5-one (edaravone), an effective free radical scavenger, provides neuroprotection in stroke models and patients. In this study, we investigated its neuroprotective effects in a chronic rotenone rat model for Parkinson's disease. Here we showed that a five-week treatment with edaravone abolished rotenone's activity to induce catalepsy, damage mitochondria and degenerate dopamine neurons in the midbrain of rotenone-treated rats. This abolishment was attributable at least partly to edaravone's inhibition of rotenone-induced reactive oxygen species production or apoptotic promoter Bax expression and its up-regulation of the vesicular monoamine transporter 2 (VMAT2) expression. Collectively, edaravone may provide novel clinical therapeutics for PD.

## Introduction

Parkinson's Disease (PD), characterized by tremor, rigidity, bradykinesia, and postural instability, is the second most common neurodegenerative disease which affects 1.7% of population aging sixty-five or elder [1], [2]. Although the pathogenesis of PD is not completely understood, environmental and genetic factors are believed to play important roles [2], [3], [4]. Subcellularly, reactive oxygen species (ROS) over-generation, oxidative stress and mitochondria dysfunction are well recognized in the pathogenesis of PD [3], [5].

Edaravone is a powerful free radical scavenger that has been clinically used to reduce the neuronal damage following cerebral ischemic stroke in Japan and China [6]. Neuroprotective effects of edaravone are shown in neonatal hypoxic-ischemic encephalopathy [7], acute intracerebral hemorrhage [6], [8], subarachnoid hemorrhage [9], amyotrophic lateral sclerosis [10], traumatic brain injury [11], [12] either animal models or patients. In all of these diseases, free radicals contribute to neuronal death. In PD, protein aggregation further generates cellular stresses that can initiate or feed into pathways to cell death evoked by oxidative stress [13]. These results illumine that edaravone may protect dopaminergic (DA) neurons and slow down the neurodegeneration in PD through anti-oxidative mechanisms.

Previous studies showed the protective effects of edaravone in 6-hydroxydopamine (6-OHDA)-treated rodent [14] and 1-methyl-4-phenyl-1,2,3,6-tetrahydropyridine (MPTP)-induced murine parkinsonian animal models [15]. However, it was reported that edaravone only reduced MPTP neurotoxicity in substantia nigra (SNc) but not in striatum (CPu) in a parkinsonian mouse model [15]. On the other hand, edaravone exerted neuroprotective effects on the whole nigrostriatal DA systems (SNc and CPu) in a 6-OHDA-induced rat model [14]. Although edaravone is involved in the anti-apoptotic, anti-oxidative and anti-inflammatory pathways in the 6-OHDA-induced parkinsonian rodent model [14], the detailed mechanisms underlying the neuroprotective effects of edaravone have not been completely understood.

In this study, our aims were to further examine the effect of edaravone in chronic rotenone-induced parkinsonian rodent animals, and utilize the findings to further characterize the neuroprotective mechanisms of edaravone by accessing PD-associated risk factors including Bcl-2 family regulation, ROS generation, SNc ultrastructure, peripheral pathological changes, and VMAT2 expression level.

## Materials and Methods

### Drugs and chemicals

Rotenone, chloral hydrate, 3,3′-diaminobenzidine, Glutaraldehyde, hematoxylin and eosin were purchased from Sigma (St. Louis, MO, USA). Triton-X100, paraformaldehyde and dimethyl sulfoxide (DMSO) were from Amresco (Solon, OH, USA). Edaravone was a gift from Simcere pharmaceuticals (Nanjing, China); Bcl-XL and Bax mouse anti-human monoclonal antibody and horseradish peroxidase-conjugated goat anti-mouse IgG secondary antibody were obtained from Santa Cruz (Santa Cruz, CA, USA). Peroxidase-conjugated streptavidin, FITC-conjugated goat-anti-mouse IgG and Cy3-conjugated goat-anti-rabbit IgG were ordered from Millipore (Billerica, MA, USA). TRIzol reagent, Superscript II reverse transcriptase (Qiagen) oligo(dT), SYBR Green PCR Master Mix, Bovine serum albumin, 2′,7′-dichlorfluorescein-diacetate (DCFH-DA) and collagenase II were from Invitrogen (Carlsbad, CA, USA). Other reagents were of analytical grade and procured locally.

### Animals Management

The experiments described in this paper were approved by the Ethical Committee on Animal Experimentation of Tongji Medical College, Huazhong University of Science and Technology, China (HUST, Approval ID: Y20080256). A total of 65 six-week-old male Wistar rats (200–250 g, from the Center of Experimental Animals, Tongji Medical College, HUST, China) were used. The animals were maintained under standard conditions with 12-hour light/dark cycles and 22±2°C and 60±5% humidity. They had access to standard laboratory chow and acidified water *ad libitum.* Rats were randomly assigned to 3 groups: 1) rotenone group (Rot-group), subcutaneous (SC) infusion of rotenone at 2 mg/kg body wt/day (for 5 weeks) mixed with soybean oil and intraperitoneal (IP) administration of 5 ml/kg saline (for 5 weeks, n = 27); 2) edaravone group (Eda-group), SC infusion of rotenone at 2 mg/kg body wt/day (for 5 weeks) mixed with soybean oil and IP administration of edaravone (2 mg/ml in saline) 10 mg/kg body wt/day (for 5 weeks, n = 22); 3) Vehicle group (Veh-group), SC injection soybean oil only in a dose of 1 ml/kg body wt/day (for 5 weeks) and IP administration of 5 ml/kg saline (for 5 weeks, n = 16). For the qRT-PCR study, TH/VMAT2 and TH/SNCA double-staining study, eight more rats of Edaravone-group and eight more rats of Vehicle-group were used.

A half of edaravone dose was injected at 7 am and another half at 7 pm. Rotenone, oil and edaravone were injected based on the rat's updating body weight until they were sacrificed for the experiment. Their body weight was recorded every day to evaluate their health conditions. Ringer lactate 1 ml was injected when they showed sign of dehydration. They were fed with Nutrison milk powder 5 g in water twice daily when they stopped eating by themselves. Specifically, the experimental timeline with number of rats used was shown in Figure S4.

### Behavioral test (Catalepsy test)

The catalepsy set-up consisted of a vertical grid and a horizontal bar to ascertain inert or static behavior. The tests were carried out between 9 am and 3 pm, always in the same context and under standard conditions [16]. All behavioral tests were taken in daylight and rats were trained for 2 days in order to prevent anxiety and unwanted fear. All rats were tested for catalepsy five weeks after rotenone or vehicle infusion. 1) **Grid test** [16], [17], [18]: Gridiron of 30 cm wide and 35 cm high with a space of 1.2 cm between each wire was used. Each rat was hung by all four paws on the vertical grid and stopwatch was started as the rat was held on grid. Stopwatch was stopped and time taken by the rats was noted as descent latency. Maximum descent latency time was fixed 180 seconds. **2) Bar test** [16], [19], [20]: rats were placed with both fore paws on a bar, which was 10 cm above the surface in half rearing position. Stopwatch started as the rat was placed on the bar and time noted as the rat removed one paw from the bar. Maximum descent latency time was fixed as 180 seconds.

### Detection of ROS

After behavioral test, rats (10 from ROT-group, 8 from Eda-group and 6 from Veh-group) were sacrificed directly and the midbrains were micropunched, intracellular ROS levels were subsequently measured. Formation of ROS was determined after a modification of the procedure described previously [21], [22], [23]. ROS detection is based on the incubation of the single cells with the nonfluorescent probe DCFH-DA, which diffuses passively through the cellular membrane. With intracellular esterase activity, DCFH-DA forms a nonfluorescent compound DCFH, which can be oxidized to the fluorescent compound DCF by ROS. In brief, small sections of midbrain tissues were micropunched, transferred into separate tubes, and digested with 0.2% collagenase II for 30 min at room temperature. After carefully removing collagenase II, Buffer solution (2 ml, 0.01 M 0.01 M sterilized phosphate buffered solution (PBS), plus 2 mM EDTA and 0.5% bovine serum albumin) was then added, and filtrated with a microfilter (30 µm). The filtrates were centrifuged (894 *g* at 4°C for 10 min), the supernatants removed completely, followed by addition of 1 ml of the buffer solution with DCFH-DA (10 mM) and incubation at 37°C for 15 min. All simples were examined for fluorescence expression using flow cytometry (BD, Franklin Lakes, NJ, USA) with an excitation wavelength of 480 nm and emission wavelength at 505–530 nm along with their negative control.

### Immunohistochemistry staining

Brains (10 from ROT-group, 8 from Eda-group and 6 from Veh-group) were removed and placed in 4% paraformaldehyde solution for 24 h. Coronal sections (5-µm) were cut from −4.5 to −6.2 mm caudal to the bregma (SNc) by using a sledge microtome (Wetzlar, Germany) [24]. The sections were de-waxed, hydrated, and the endogenous peroxidase was quenched with 0.3% H_2_O_2_ for 30 min. After antigen retrieval, the slides were treated with 0.5% Triton-X100 for 30 min and 5% bovine serum albumin for 30 min. The sections were first incubated overnight with antibody/phosphate buffered solution (PBS; Bcl-xl, or Bax antibody, or tyrosine hydroxylase (TH, mouse monoclonal antibody, Millipore, Billerica, MA, USA) antibody 1∶100 dilution) at 4°C and then incubated with FITC-conjugated goat-anti-mouse IgG or the secondary biotinylated goat anti-rabbit IgG or for 60 min and with peroxidase-conjugated streptavidin for 45 min. The immunoreactions were visualized by 3,3′-diaminobenzidine for 15–20 min. The pictures were densitometrically observed and analyzed by employing the Image-Pro plus 6.0 software package (Media Cybernetics Inc, Bethesda, MD, USA).

For the TH/VMAT2 and TH/SNCA double-staining, four more rats of each group were intraperitoneally injected with edaravone (10 mg/kg, edaravone: DMSO  = 9∶1, Edaravone-group) or vehicle (PBS : DMSO  = 9∶1, Vehicle-group) twice in 24 h. Paraformaldehyde perfusion was used to sacrifice all the rats. Embedded with OCT compound, brain tissues were cut into serial 10-µm-thick slices with a cryostat. Immunofluorescent staining was employed to label the TH (Millipore, mouse monoclonal antibody) and VMAT2 (rabbit polyclonal antibody, Sigma-Aldrich), or TH (Millipore, rabbit monoclonal antibody) and SNCA (BD, mouse monoclonal antibody) in the SNc (from −4.5 mm to −6.2 mm caudal to bregma). Expression was visualized by the CY3-conjugated goat-anti-rabbit IgG, FITC-conjugated goat-anti-mouse IgG (Millipore). For counting TH-Positive Cells and densitometrically analyzing VMAT2 expression, a design-based unbiased stereological method and a morphometry / image analysis system (MacBiophotonics ImageJ, McMaster Biophotonics Facility, Hamilton, ON, USA) was used as previously described [25], [26], [27].

### Ultrastructure of the SNc

Rats (7 from ROT-group, 6 from Eda-group and 4 from Veh-group) were deeply anesthetized after behavioral test and perfused through the aorta with ice-cold PBS and then with paraformaldehyde (4% wt/vol) and glutaraldehyde (1% wt/vol) in PBS [28]. Brains were post-fixed in 2.5% glutaraldehyde at 4°C for 6 h after the separation. A 1-mm^3^ tissue block from the left and right SNc regions (−4.5 to −6.2 mm caudal to the bream) was micropunched, fixed in PBS containing 2.5% glutaraldehyde, and preserved at 4°C for further processing. The fragments were post-fixed in 1% osmium tetroxide in the same buffer, dehydrated in graded alcohols, embedded in Epon 812, sectioned with an ultramicrotome, and stained with uranyl acetate and lead citrate. The sections were then examined under a transmission electron microscope (TEM; Technai 10, Philips, Netherlands).

### Pathology of the peripheral organs

The pathological evaluation of the peripheral organs was performed on the some rats as immunohistochemistry study (10 from ROT-group, 8 from Eda-group and 6 from Veh-group). The animals were deeply anesthetized and perfused through the aorta with ice-cold PBS and then with paraformaldehyde (4% wt/vol) in PBS. The heart, kidney, liver, lung, spleen, and stomach were taken out and immersed in 4% paraformaldehyde in PBS at 4°C for 24 h before paraffin-embedding. The tissue sections were cut into 5-µm pieces, stained with hematoxylin and eosin (HE), and examined under a microscope (B51, Olympus, Tokyo, Japan).

### Quantification of VMAT2 mRNA levels by qRT-PCR

To further study the neuroprotective mechanism of edaravone on cell and rats, four more rats of each group were intraperitoneally injected with edaravone (10 mg/kg, edaravone: DMSO  = 9∶1, Edaravone-group) or vehicle (PBS: DMSO  = 9∶1, Vehicle-group) twice in 24 h. Live decapitation was used to sacrifice all the rats, and left and right SNc regions (−4.5 to −6.2 mm caudal to the bregma) were micro-punched. The tissues were added into RNase-free 1.5 ml Microfuge tubes with 100 µl TRIzol reagent, and then homogenized in ice by mechanical trituration, followed by additional 900 µl TRIzol reagent added into the tubes for isolating total RNA. Total RNA extraction from brain tissues, reverse transcription and quantitative PCR reactions methods have been described previously [29], [30]. Briefly, reverse transcription was performed with Superscript II reverse transcriptase (Qiagen) oligo(dT) as the primer. Quantitative PCR reactions were conducted with SYBR Green PCR Master Mix and primers listed in Figure S1. Glyceraldehyde-3-phosphate dehydrogenase (GAPDH) and β-actin were used as internal controls. Relative expression levels were calculated by using the 2^-ΔΔCT^ method. VMAT2 and SNCA primers showed amplification efficiency approximately equal (within 5% difference) to that of the internal controls.

### Statistical analyses

The statistical analyses were carried out using SPSS version 12.0 for Windows (SPSS, Chicago, IL, USA). As all groups showed normal distribution, intergroup differences were assessed by one-way analysis of variance (ANOVA) and/or Student's *t*-test. The results were presented as the means ± SD. The *P* value <0.05 was considered statistically significant.

## Results

### Edaravone conspicuously shortened the descent latency in rotenone-treated rats

Five weeks after rotenone administration, 22 of 27 (81.48%) Rot-group rats developed characteristic behavioral features of PD, such as back hunching and stiffness, face-washing behaviors, bradykinesia, or hypokinesia [24]. In these 22 Rot-group rats, 4 rats were fed with Nutrison milk powder 5 g in water twice daily when they stopped eating by themselves, while other 23 Rot-group rats had acceptable health conditions (self-catering, self-cleaning and self-defecation observed). No typical behavioral feature of PD or other health problems were observed in Eda-group and Veh-group (The chemical structure of edaravone was shown in Figure S2).

**Figure 1:**
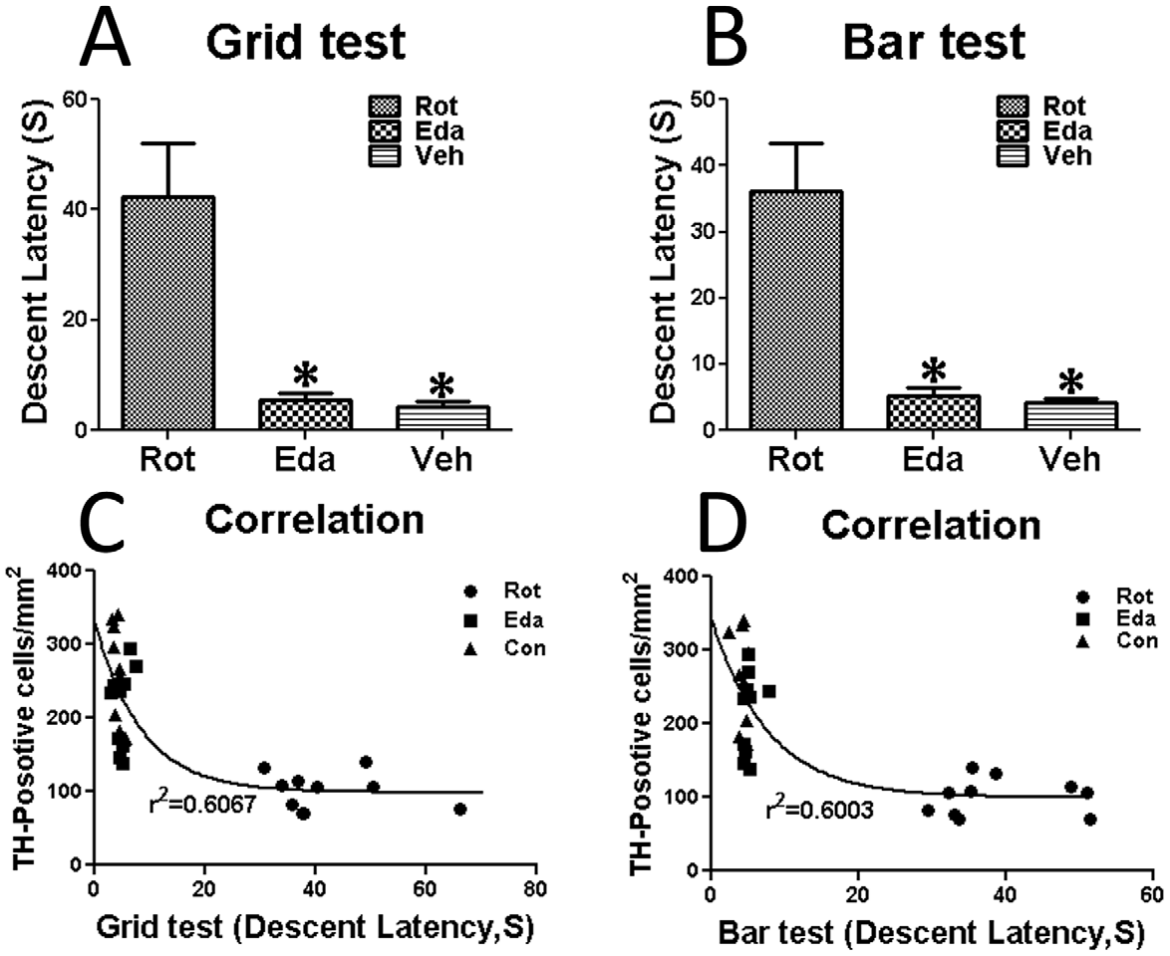
Catalepsy test of experimental animals (five weeks after the first administration of drugs). (A), (B), descent latency of Rot-, Eda- and Veh-group by Grid test and Bar test, respectively (*P<0.05 as compared to Rot-group); (C), correlation between SN TH-positive cell numbers and Grid test scores; (D), correlation between SN TH-positive cell numbers and Bar test scores.

For further accessing the behavioral profile of these animals, prolonged descent latency was found in Rot-group as compared to Eda-group and Veh-group by Grid-iron test (Fig. 1A). Bar Test also showed descent latency in Rot-group was quite longer than Eda-group and Veh-group (Fig. 1B). Edaravone respectively reduced 87.17% and 85.37% the prolongation of descent latency of Grid test and Bar test (Fig. 1). Moreover, there was no significant difference between Veh-group and Eda-group, suggesting that edaravone significantly ameliorated the rotenone-induced behavioral defects.

### Edaravone significantly prohibited rotenone-induced ROS excessiveness in midbrain

**Figure 2:**
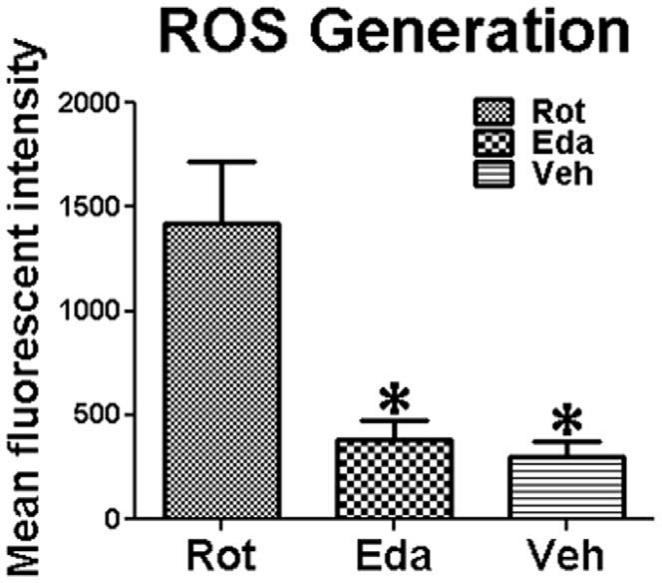
Effect of Edaravone on Rotenone-induced ROS generation in midbrain. Fluorescent intensity in midbrain of DCF, the product of cellular oxidation of DCFH-DA, as detected by flow cytometry for Rot-, Eda- and Veh-group (*P<0.05 as compared to Rot-group).

Flowcytometric data showed higher ROS generation in midbrain of Rot-group than that in Eda-group and Veh-group (Fig. 2). Edaravone inhibited 73.35% of rotenone-induced ROS generation in midbrain tissues. There was no significant difference between Eda-group and Veh-group midbrain tissues in ROS generation, suggesting edaravone is a powerful free radical scavenger.

### Edaravone protected DA neurons in SNc and DA terminal in CPu

**Figure 3:**
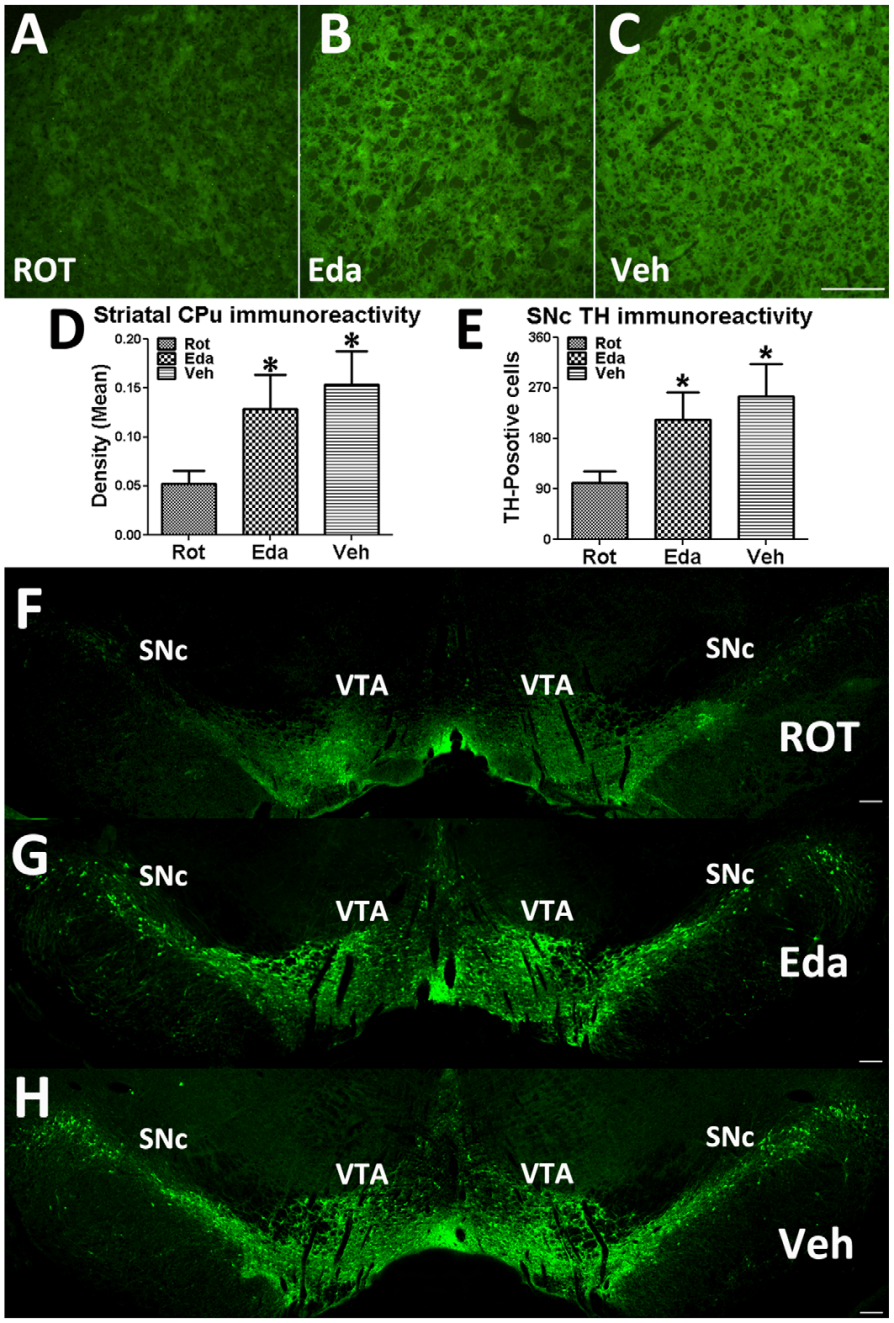
TH immunoreactivity in CPu and SNc. (A), (B) and (C), TH immunoreactivity in the CPu of Rot-group, Eda-group and Veh-group, respectively; (F), (G) and (H), TH-positive neurons in the SNc of Rot-group, Eda-group and Veh-group; (D) and (E), Quantitation of TH immunoreactivity in CPu and TH-positive cells in SNc (* P<0.05 compared to ROT-group; scale bars  = 100 µm).

**Figure 4:**
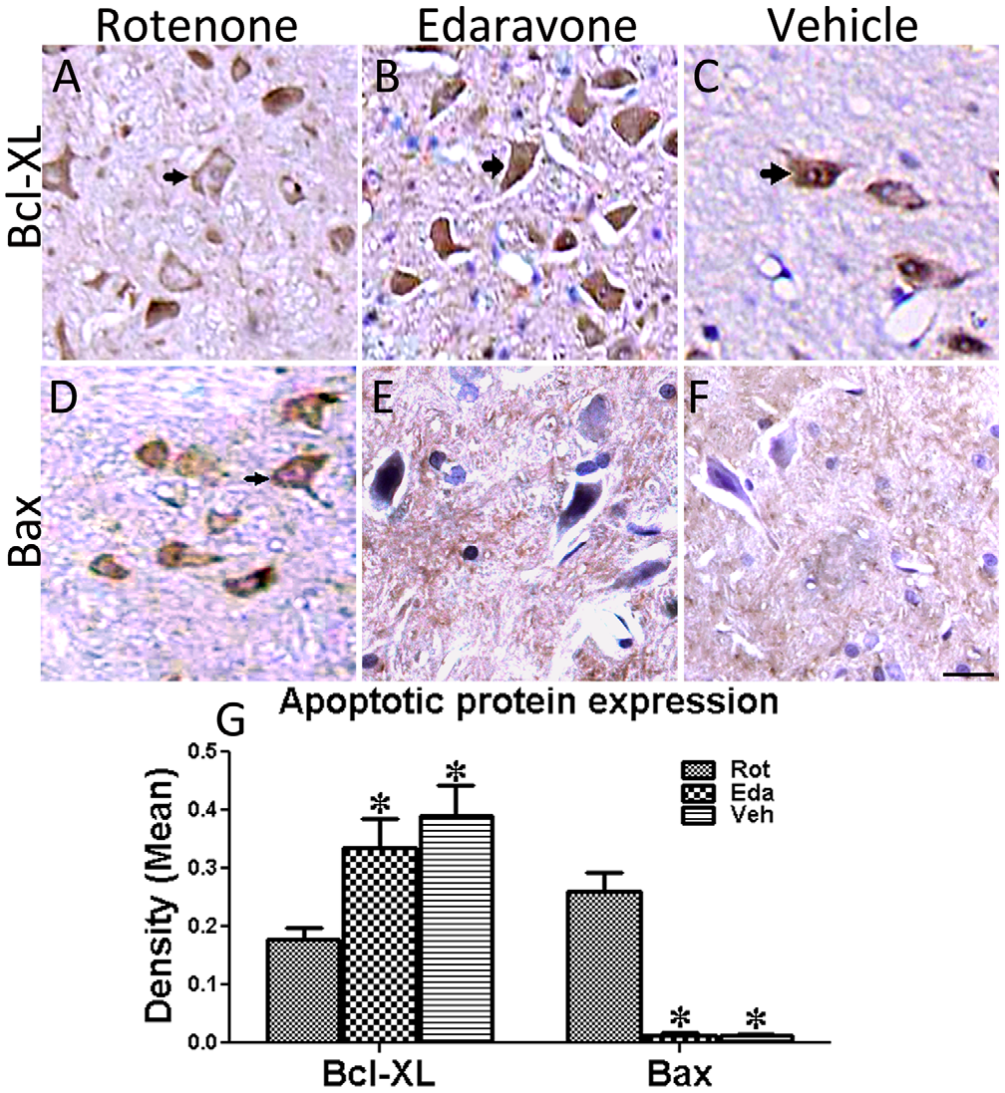
Anti-apoptosis effects of Edaravone on Rotenone-induced animals. Bcl-XL and Bax immunostaining of the SNc from all groups five weeks after behavioral test (10 from ROT-group, 8 from Eda-group and 6 from Veh-group). (A),(B) and (C), Bcl-XL expression in Rot-, Eda- and Veh-group respectively; (D),(E) and (F), immunohistostaining of Bax in all these three groups; (G) quantitative analysis of Bcl-XL and Bax expression in all these three groups, respectively. (Scale bars  = 20 µm, *P<0.05 as compared to Rot-group).

Histological examination of brain tissues indicated that rotenone infusion significantly decreased (by 65.67%) integrated intensity of TH immunostaining in CPu (Fig. 3A, C and D), while the TH staining intensity was only decreased by 16.10% in the CPu of Eda-group compared to the CPu of Veh-group (Fig. 4B, C and D). The death rate of TH-positive cells in SNc was 60.48% and 15.82% in Rot-group and Eda-group respectively (Fig. 3E–H). Edaravone rescued 44.66% of dopamine neurons from rotenone-induced degeneration. Moreover, the correlation study on SN degeneration *versus* behavioral scores indicated that there was a significantly negative relationship between TH-positive cell numbers and the descent latency of Grid test (Fig. 1C) or Bar test (Fig. 1D), although this correlation could be attributed partly to peripheral toxicity as well.

### Edaravone inhibited apoptotic protein Bax expression in rotenone-treated rats

After behavioral test, eight randomly chosen rats of each group were used for immunohistostaining of anti-apoptotic protein Bcl-XL and pro-apoptotic protein Bax. The immunohistological examination of the SNc indicated that the rotenone infusion significantly down-regulated the expression Bcl-XL (Fig. 4A) and up-regulated the expression of Bax (Fig. 4D) in the SNc of Rot-group. However, edaravone conspicuously attenuated rotenone-induced down-regulation of Bcl-XL (Fig. 4B). Interestingly, Bax positive cells were not observed in the SNc of Eda-group and Veh-group rats. The quantitative analysis demonstrated that Bcl-XL expression was decreased by 54.36% (Fig. 4A,C,G), as compared to Veh-group. There was no significant difference in Bcl-XL and BAX expression between Eda-group and Veh-group (Fig. 4B,C,E,F), indicating the predominant anti-apoptosis effects of edaravone on rotenone-treated animals.

### Edaravone ameliorated rotenone-induced mitochondrial damage in the substantia nigra

**Figure 5:**
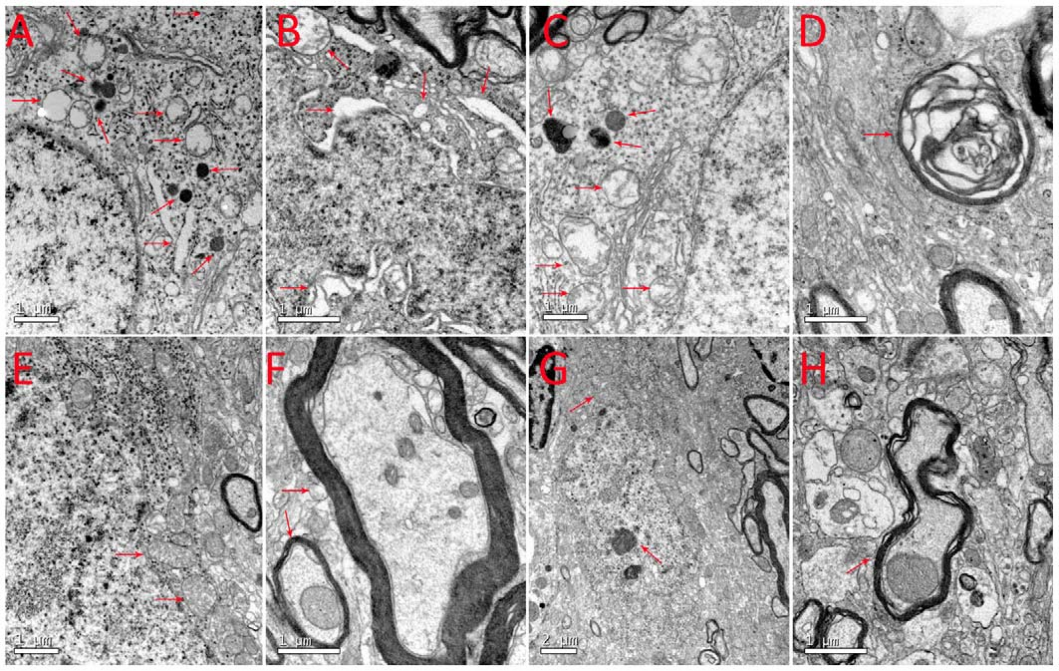
Ultrastructural changes in SNc. (A): mitochondrial swelling, mitochondrial crest fracture, mitochondrial vacuolar degeneration, dilated and broken rough endoplasmic reticula, liberation of ribosomes from the rough endoplasmic reticula and the increased formation of lysosomes; (B): mitochondrial swelling, mitochondrial crest fracture, dilated and broken rough endoplasmic reticula, perinuclear space augmentation; (C): mitochondrial swelling, mitochondrial crest fracture, the increased formation of secondary lysosome formation;(D): degeneration of the medullary sheathes; (E, G): normal mitochondria, rough endoplasmic reticulum and ribosomes in the SNc neurons of the Veh-group and Eda-group rats; (F, H): normal medullary sheathes from the Veh-group and Eda-group rats (7 rats from ROT-group, 6 rats from Eda-group and 4 rats from Veh-group).

The left and right SNc (from −4.5 to −6.2 mm caudal to the bregma) of the all groups were micro-punched and examined under a transmission electron microscope. There was no ultrastructural difference between the SNc of Eda-group (Fig. 5G,H) and Veh-group (Fig. 5E,F), while mitochondrial swelling, mitochondrial crest fracture, mitochondrial vacuolar degeneration, dilated and broken rough endoplasmic reticula, liberation of ribosomes from rough endoplasmic reticula, the increased formation of primary and secondary lysosomes, and perinuclear space augmentation were observed in SNc neurons of Rot-group (Fig. 5A–C). Medulla sheathes degeneration was also detected in the Rot-group (Fig. 5D), while normal Medulla sheathes were observed in Eda-group (Fig. 5H) and Veh-group (Fig. 5F).

### Edaravone up-regulated VMAT2 expression without affecting SNCA expression

**Figure 6:**
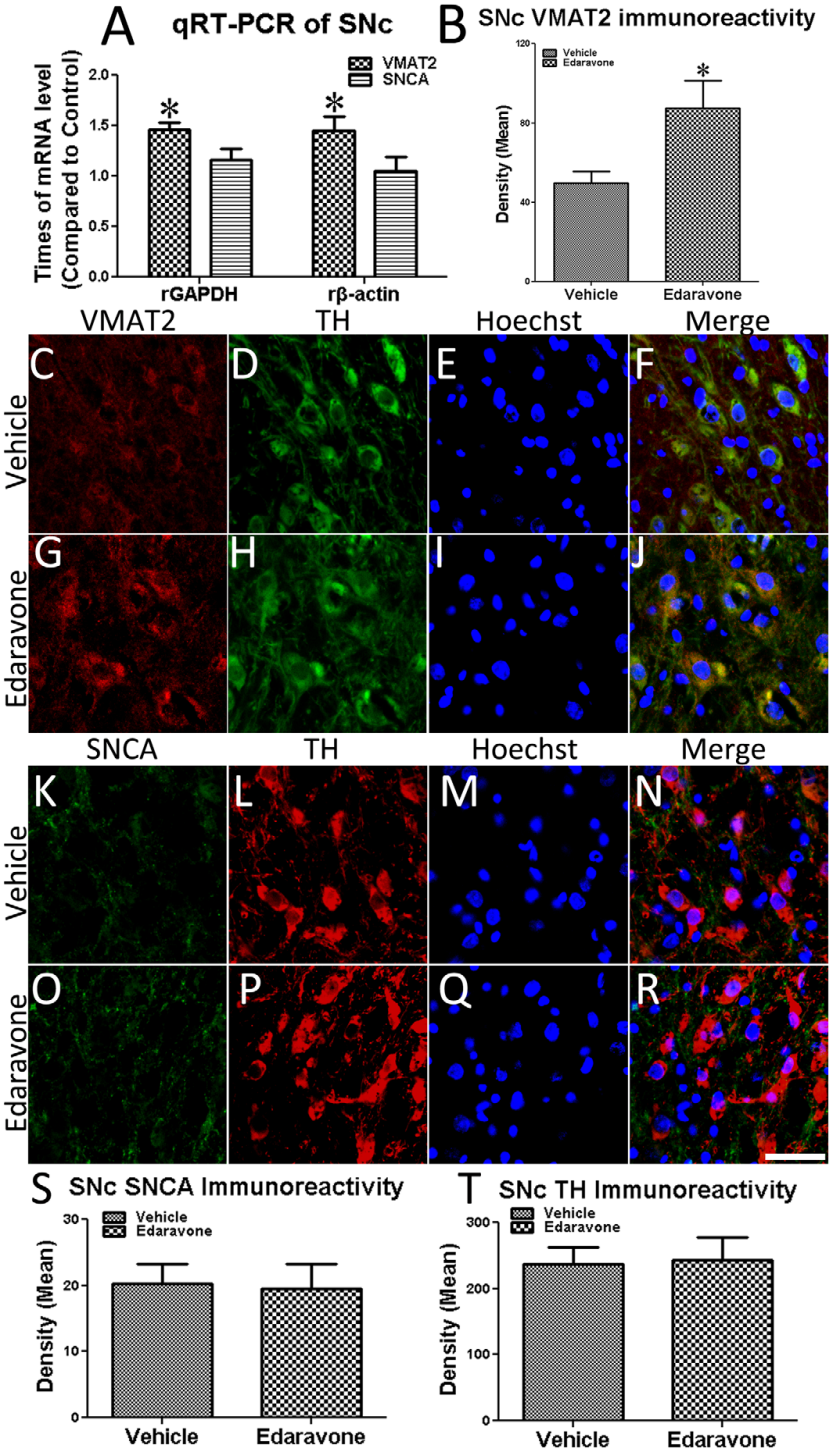
Edaravone increased mRNA level of *VMAT2* in rats SNc. (A) mRNA levels of VMAT2 and SNCA in edaravone-infused SNc tissue compared to Veh-group (* P<0.05 compared to Veh-group); (B) Quantification of VMAT2 immunostaining; (C–F) and (G–J), double-immunostaining of TH/VMAT2 in the SNc of Vehicle rats and edaravone rats, respectively; (K–N) and (O–R), double-immunostaining of TH/SNCA in the SNc of Vehicle rats and edaravone rats, respectively; (Scale bar  = 50 µm); (S) Quantification of SNCA immunostaining; (T) Quantification of TH-positive cell numbers.

In rat midbrain SNc tissues, edaravone infusion up-regulated the VMAT2 mRNA levels by 45.83% (controlled by GAPDH) and 44.93% (controlled by β-actin, Fig. 6A) in Edaravone-group compared to that in Vehicle-group. Double-immunostaining of TH/VMAT2 showed that VMAT2 protein expression in the DA neurons of the edaravone rats was up-regulated by 69.94% compared to the Vehicle-treated rats (Fig. 6B–J). SNCA mRNA levels (Fig. 6A), SNCA protein expression (Fig. 6K–S) and TH-positive cell numbers (Fig. 6L,P,T) in the SNc displayed no difference between Eda-group and Veh-group, suggesting that NBP up-regulated the expression of the DA neurons-protective protein VMAT2 without inducing SNCA over-expression (which has been proven to be harmful to DA neurons).

### Edaravone prevented rotenone-induced toxicity in peripheral organs

**Figure 7:**
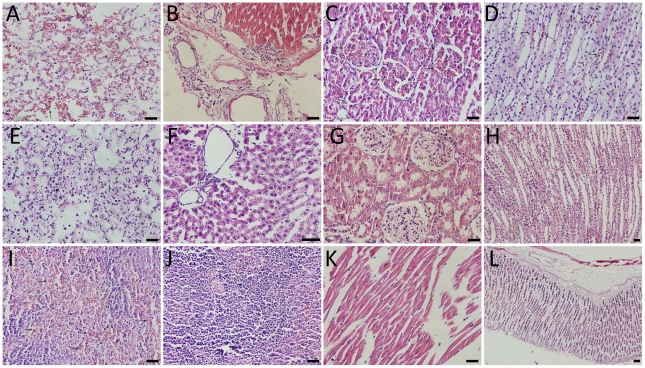
Pathological changes in peripheral organs of Parkinsonian rats. (A): Neutrophilic infiltration in the pulmonary alveoli; (B): neutrophil infiltration in the periportal areas, the expansion of central veins and bile ducts; (C) and (D): Red blood cells were increased in the renal glomeruli and renal medulla; (E): The normal alveolar walls were thin and delicate; (F): the normal hepatic lobules and central veins; (G) and (H): the normal renal glomerulus and renal medulla; (I): hemorrhage and hemosiderin deposition in the spleen of Rot-group rats; (J): the normal white pulp of the spleen; (K) and (L): normal heart and stomach of Rot-group rats (10 rats from ROT-group, 8 rats from Eda-group and 6 rats from Veh-group; Scale bars  = 50 µm).

No animals died spontaneously within five weeks of the systemic rotenone administration (2.0 mg/kg/day). There were no pathological changes observed in the peripheral organs of Eda-group and Veh-group rats. However, significant pathological changes were observed in the liver, kidney, lung, and spleen, while there was no obvious change in the heart or stomach in Rot-group rats. In Rot-group, neutrophilic infiltration were detected in the lung (Fig. 7A), while the alveolar walls of the Eda-group and Veh-group rats were thin and delicate (Fig. 7E). In the liver, neutrophil infiltration in the periportal areas, the expansion of central veins and bile ducts were observed in the Rot-group (Fig. 7B), but they were intact in the Eda-group and Veh-group (Fig. 7F). In the kidney, the number of red blood cells increased in the renal glomeruli (Fig. 7C) and renal medulla (Fig. 7D), while few red blood cells were seen in the renal glomeruli of Eda-group and Veh-group (Fig. 7G,H). In the spleen, hemorrhage and hemosiderin deposition were observed in the Rot-group (Fig. 7I), while white pulp was noted in the spleen in Eda-group and Veh-group animals (Fig. 7J). Rotenone exerted little influence on the heart (Fig. 7K) and stomach (Fig. 7L) in Rot-group rats.

### Edaravone prohibited rotenone-induced body weight loss in animals

Our data showed the body weight of Rot-group animals and Veh-group animals were significantly different at all time points except on day 0 (P<0.05, Figure S3). However, there was no significantly difference between the body weight of Eda-group animals and Veh-group animals in the whole period of 5 weeks (Figure S3). Moreover, no animals developed constipation (data not shown). However, stool frequency was transiently decreased in rotenone-treated rats during the first 12 days of rotenone exposure and then recovered to control values, which could be associated with the body weight loss of these rats during the first 12 days of rotenone administration.

## Discussion

We have shown that the main pharmacologic features of edaravone's effective neuroprotection include: 1) attenuation of rotenone-induced characteristic parkinsonian behaviors in rats; 2) prevention of rotenone-induced over-generation of ROS in midbrain; 3) inhibition of apoptotic protein Bax expression in the SNc of rotenone-treated rats; 4) prevention of rotenone-induced pathological changes in peripheral organs; 5) protection of mitochondria in SNc neurons against rotenone toxicity; and 6) up-regulation of VMAT2 expression.

We have previously reported that both rotenone-based stereotaxical and systemic parkinsonian rodent models could recapitulate nigrostriatal DA lesions and mimic the clinical features of idiopathic PD. They both could be used to study pathogenesis, pathology and pathophysiology of PD and to search for effective treatments for PD [24], [31], [32], [33]. However, the stereotaxical model is more suitable for long-term studies as the behavioural changes progress gradually until the 24^th^ week, while the systemic model is better for studies of both nigrostriatal system and peripheral system because of the rotenone-induced peripheral toxicity [24], [31], [32], [33]. In this study, the systemic rotenone-induced parkinsonian rodent model has been chosen for three reasons. 1) Systemic administration is easily exercisable and also highly reproducible (in our hands, 22 of 27 or 81% rats developed typical behavioral features of PD) without going through surgery [24], [31], [32], [33]; 2) this systemic model is capable of reproducing the progression of PD-like pathology while the parkinsonian symptoms could be reversed by L-DOPA [34], [35]; 3) this systemic model could be used to examine protective effects in peripheral system as well [24]. Therefore, we postulated that this systemic model helps better understand the protective activity of edaravone.

Previous studies have shown that edaravone attenuated neurotoxin-induced decreases in dopamine levels, TH immunostaining in the SNc [14], [15] and CPu [14], indicating neuroprotective effects of edaravone in PD models. However, edaravone was reported to only reduce MPTP neurotoxicity in SNc but not in CPu in parkinsonian mouse model [15]. Together with our data, the discrepancies of all these results may attribute to: different neurotoxin-based parkinsonian animal models (MPTP, 6-OHDA and rotenone), different administration route (i.p. and i.v.), different dosage of edaravone (1 and 3 mg/kg, 100 and 250 mg/kg, 10 mg/kg), different animal species (mice *versus* rat), different characteristics of the behavioral test, alteration of edaravone-activity and affinity over time after lesioning and of different detailed regimen [14].

It has been reported that edaravone exerts its neuroprotection via anti-apoptotic, anti-oxidative and anti-inflammatory pathways in the 6-OHDA-based parkinsonian model, but the detailed anti-apoptotic mechanism has not been explored. Our data have shown that edaravone up-regulated Bcl-XL expression and inhibited Bax expression in parkinsonian rats, helping understand the underlying mechanisms of edaravone's anti-apoptosis effects, similar to the findings in stroke animal models [36], [37]. Other neuroprotective mechanisms may include reduction of Fas-associated death domain protein to suppress apoptotic cell death (in cerebral infarct) [38], alleviation of dysfunction of endoplasmic reticulum with subsequent cell death (as seen in cerebral ischemia) [39], and decrease in MAP kinase activity in astrocytes [40]. The ultrastructural study demonstrated for the first time that edaravone significantly prevented mitochondrial damage and prohibited rotenone-induced typical ultrastructural changes, including mitochondrial swelling, mitochondrial crest fracture and mitochondrial vacuolar degeneration, in addition to a previous study which showed edaravone ameliorated mitochondrial respiratory function, restrain, and prevented mitochondrial membrane potential loss induced by MPP+ [41].

**Figure 8:**
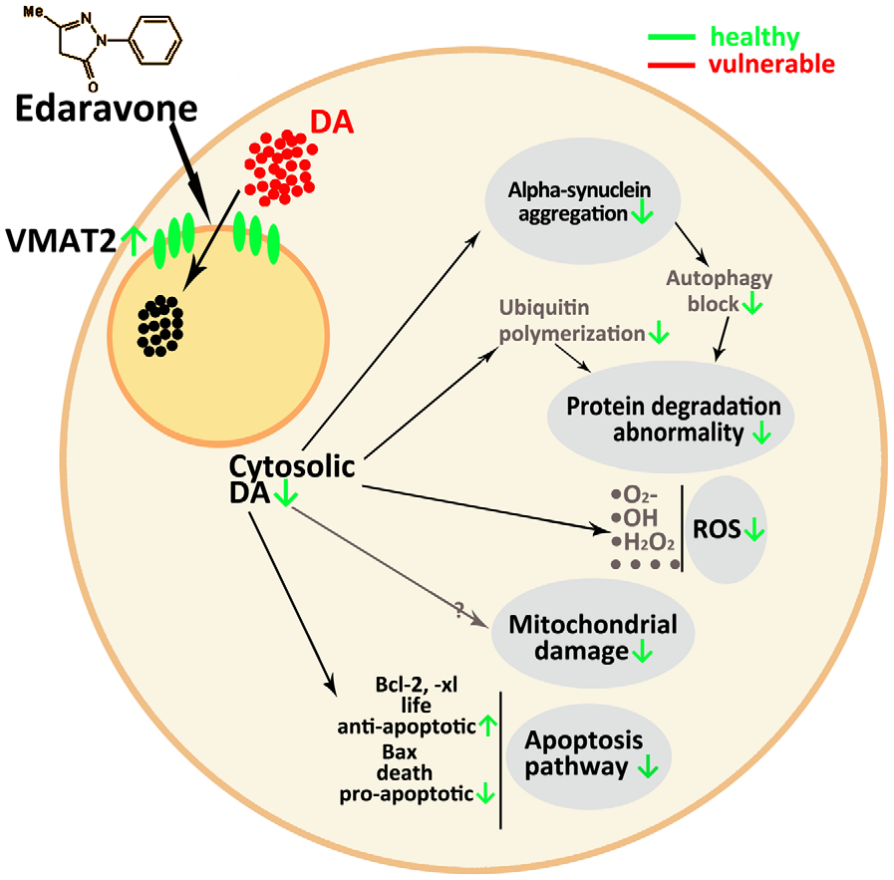
Working model for protective effect of edaravone on DA neurons. Up-regulation of VMAT2 expression by edaravone treatments to reduce cytosolic dopamine concentration, along with down-regulation of apoptosis pathways, may make DA neurons healthier.

More interestingly, we have shown that edaravone is able to up-regulate VMAT2 expression in the related brain region. VMAT2 takes up cytosolic dopamine and reduces the cytotoxicity of dopamine. Deletion of VMAT2 causes lethality in mice [42] and DNA sequence variation in the human gene is associated with PD [43], [44]. Consistently, striatal VMAT2 protein levels are reduced significantly in PD patients [45]. Moreover, rotenone inhibits VMAT2 activity and rotenone even induces VMAT2 accumulation of aggregate-like formations consequently to the redistribution of dopamine to the cytosol and apoptosis of DA cells [46]. In addition, up-regulating the expression level of VMAT2 could protect DA cells against neurotoxicity [47], [48]. Buildup of cytosolic dopamine is associated with oxyradical stress and possible dopamine interaction with PD-related proteins including SNCA and Parkin [49], [50]. Finally, cytosolic dopamine could cause protein degradation abnormality by ubiquitin polymerization and autophagy block, which contributes to protein aggregation in DA neurons [49], [51]. Therefore, increased VMAT2 expression may contribute to the neuroprotective effects (Fig. 8).

In the present study, the SC rotenone model was a chronic model, and rotenone could accumulate in the SC adipose tissue because of its lipophilic nature [24], together with soybean oil, rotenone slowly entered body fluid circulation. However, edaravone could rapidly attain the steady-state plasma drug concentration through IP injection, which might help to attenuate rotenone-induced toxicity since there is no direct interaction between rotenone and edaravone [52]. Furthermore, the peripheral organs injury in Rot-group rats could deteriorate their parkinsonian symptoms as the difficulty in eating, self-cleaning and weight losing. These might explain the significant parkinsonian symptoms in Rot-group and effective protection of edaravone on rotenone-induced SNc and peripheral organs.

In summary, by regulating multiple molecular and cellular events (Fig. 8), edaravone protects dopamine neurons as well as peripheral tissues very effectively in the rotenone models for PD. The therapeutic potentials of edaravone are supported by not only its antioxidation activity but also gene regulation (Fig. 8). Moreover, it provides anti-apoptotic effects besides anti-oxidative effects in animal models, consistent with its neuroprotection in clinical stroke patients [9], [12]. Therefore, edaravone may represent a more plausible medicine for patients with PD than other antioxidants such as vitamin [53]. Since pretreatment of edaravone is an excellent method to prevent DA neurons from degeneration in rotenone-induced parkinsonian model, it is expected that treatment of edaravone may slow down the progression of neurodegeneration in patients with PD, as observed in those with stroke.

## Supporting Information

Figure S1 **Primers for qRT-PCR.**
(TIF)

Figure S2 **The chemical structure of edaravone.**
(TIF)

Figure S3 **Effect of edaravone on Rotenone-induced body weight loss in rats.** Statistical analysis showed significant difference in body weight between Rot-group animals and Veh-group animals at all time points except on day 0 (P<0.05). No significant difference was found between Eda-group animals and Veh-group animals in the whole period of 5 weeks.
(TIF)

Figure S4 **Experimental timeline with number of rats used.**
(TIF)
