# Supplementary figures and images for: Edaravone Guards Dopamine Neurons in a Rotenone Model for Parkinson's Disease
Source: PLoS One. 2011 Jun 3;6(6):e20677. doi: 10.1371/journal.pone.0020677 (PMC3108992; doi:10.1371/journal.pone.0020677)

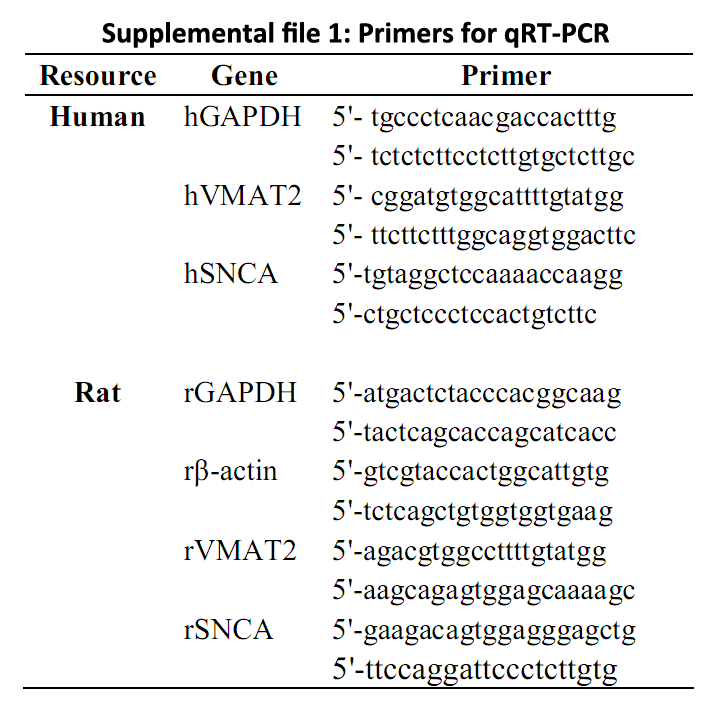

Supplement: Figure S1 — Primers for qRT-PCR. (TIF) [file pone.0020677.s001.tif]

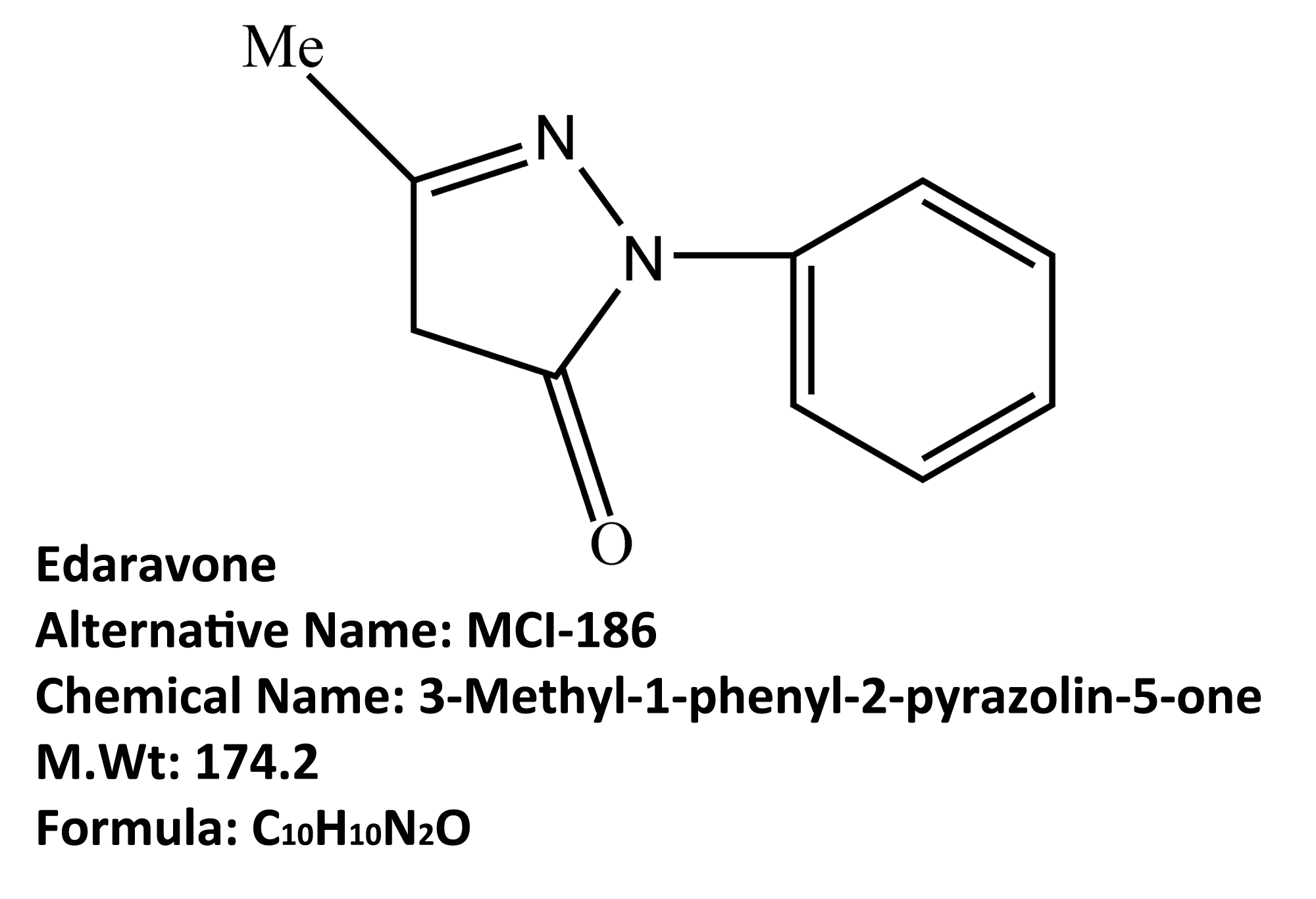

Supplement: Figure S2 — The chemical structure of edaravone. (TIF) [file pone.0020677.s002.tif]

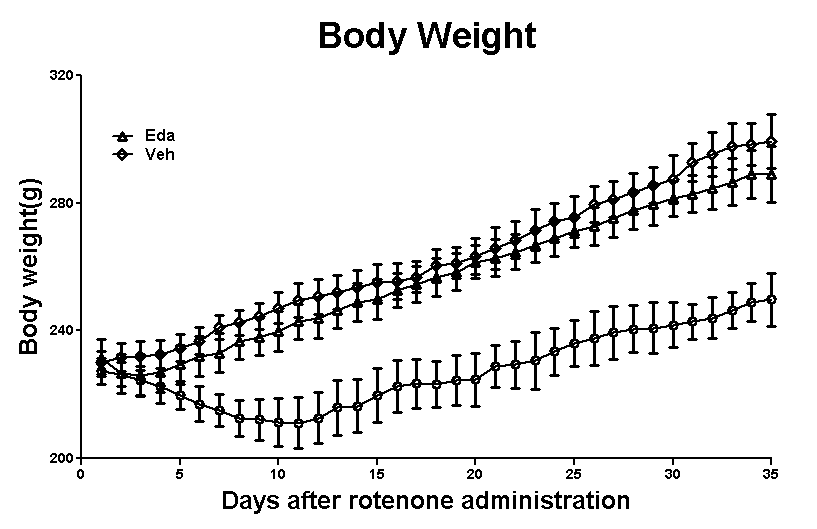

Supplement: Figure S3 — Effect of edaravone on Rotenone-induced body weight loss in rats. Statistical analysis showed significant difference in body weight between Rot-group animals and Veh-group animals at all time points except on day 0 (P<0.05). No significant difference was found between Eda-group animals and Veh-group animals in the whole period of 5 weeks. (TIF) [file pone.0020677.s003.tif]

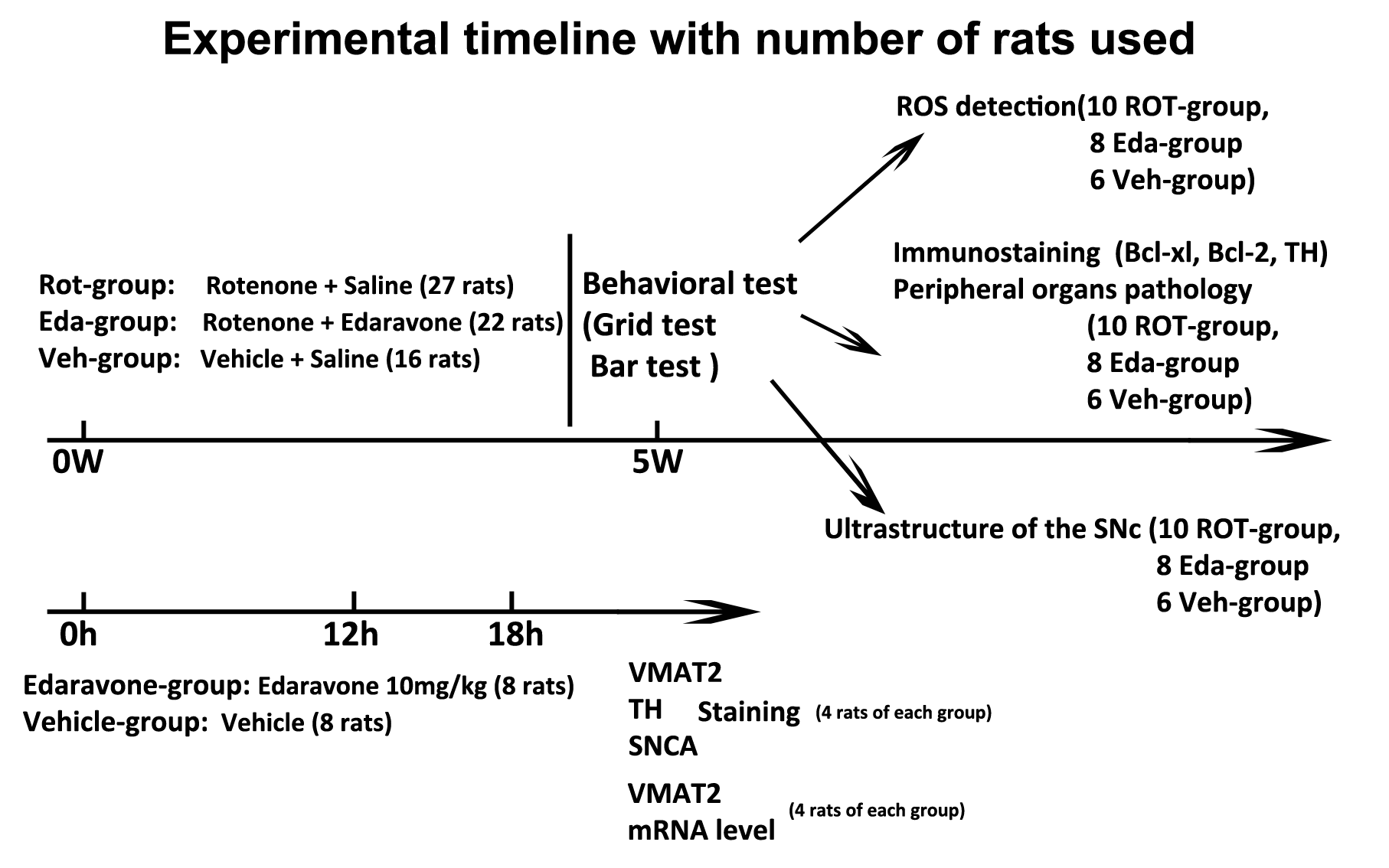

Supplement: Figure S4 — Experimental timeline with number of rats used. (TIF) [file pone.0020677.s004.tif]
